# Chronic Cholestatic Liver Disease Induced by Larval Ascariasis: Novel Insights Into Immune‐Mediated Pathogenesis and Hepatic Fibrosis in Mice

**DOI:** 10.1096/fj.202600771R

**Published:** 2026-05-17

**Authors:** Jorge Lucas Nascimento Souza, Chiara Cássia Oliveira Amorim, Ana Rafaela Antunes‐Porto, Fernanda Rezende Souza, Evelyn Ane Oliveira, Izabela da Silva Oliveira, Isabela de Brito Duval, Andressa Mariana Saldanha‐Elias, Marcelo Eduardo Cardozo, Ramayana Morais de Medeiros Brito, Luisa Mourão Dias Magalhães, Geovanni Dantas Cassali, Neima Briggs, Ricardo Toshio Fujiwara, Remo Castro Russo, Guilherme Grossi Lopes Cançado, Lilian Lacerda Bueno

**Affiliations:** ^1^ Laboratory of Immunobiology and Control of Parasites, Department of Parasitology, Institute of Biological Sciences Universidade Federal de Minas Gerais Belo Horizonte Brazil; ^2^ Laboratory of Pulmonary Immunology and Mechanics, Department of Physiology and Biophysics, Institute of Biological Sciences Universidade Federal de Minas Gerais Belo Horizonte Brazil; ^3^ Laboratory of Comparative Pathology, Department of Pathology, Institute of Biological Sciences Universidade Federal de Minas Gerais Belo Horizonte Brazil; ^4^ Laboratory of Immunology of Parasitic Interactions, Department of Parasitology, Institute of Biological Sciences Universidade Federal de Minas Gerais Belo Horizonte Brazil; ^5^ Department of Internal Medicine (Infectious Diseases) Yale University School of Medicine New Haven Connecticut USA; ^6^ Instituto Alfa de Gastroenterologia, Hospital das Clínicas Universidade Federal de Minas Gerais Belo Horizonte Brazil

**Keywords:** chronic inflammation, comorbidities, helminth infection, larval ascariasis, murine model, tissue adaptation

## Abstract

Ascariasis is a widespread helminthic infection, yet the long‐term hepatic consequences of larval migration remain poorly understood. Evidence indicates that tissue damage may persist beyond parasite clearance. While chronic cholestatic liver disease is classically associated with obstructive, autoimmune, or genetic disorders, parasitic infections such as ascariasis may represent an overlooked etiology. This study investigated post‐migratory hepatic effects of *Ascaris suum* larvae in a mouse model, evaluating dose‐dependent and exposure‐frequency paradigms. Female BALB/c mice were infected with *A. suum* eggs using low‐dose (250 eggs) or high‐dose (2500 eggs), administered as single or reinfection (14 days apart). Mice were euthanized at three time points, day 4 post‐infection (dpi) (peak hepatic migration phase), or 35 and 100 dpi (post‐hepatic clearance). Livers were analyzed for parasite burden (4 dpi), cytokine gene expression, and comprehensive histopathological analysis (35/100 dpi). Plasma was assessed for biochemical markers (AST, ALT, GGT, ALP, total bilirubin and fractions, and albumin) and cytokine quantification. Histological analysis revealed persistent hepatic inflammation and fibrotic remodeling up to 100 dpi. Biochemical assays confirmed cholestatic dysfunction with elevated ALP, GGT, and bilirubin. Gene expression analysis showed sustained inflammatory signaling. These findings establish that larval migration induces chronic hepatobiliary injury and cholestasis, independent of peak burden. Importantly, pathology occurred without adult worm–mediated biliary obstruction, identifying a novel helminth‐driven hepatic disease mechanism. Our findings demonstrated that ascariasis, even at low infection intensities and in the absence of adult worm establishment, can induce persistent hepatic inflammation, fibrotic progression, and cholestatic dysfunction. This evidence challenges the prevailing paradigm that biliary obstruction by adult worms is the exclusive mechanism of hepatobiliary injury in ascariasis. Our results underscore the significant pathogenic potential of larval stages and their ability to provoke chronic liver pathology that persists well beyond parasite clearance. These observations are clinically relevant for endemic regions, where recurrent low‐level exposure may lead to cumulative liver damage despite the absence of overt adult worm infection.

## Graphical abstract

Liver alterations induced by single infection (SI) or reinfection (RE) with 250 or 2500 *Ascaris suum* eggs in mice. Histological analysis shows inflammatory cell infiltration (eosinophils, lymphocytes, macrophages, and neutrophils), fibrotic areas, and bile duct proliferation in infected animals compared with uninfected controls. These alterations were associated with increased serum liver enzymes (AST, ALT, GGT, ALP) and bilirubin. The magnitude of inflammation, immune regulation, and fibrosis varied with infection dose and exposure, being most pronounced in reinfected groups, particularly RE 2500.

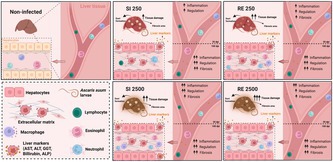

## 1 Introduction

Ascariasis is a neglected tropical disease (NTD) caused by *Ascaris lumbricoides* or *A. suum* [1, 2]. Recent global estimates indicate that ascariasis is endemic in over 150 countries, affecting nearly 450 million people globally, with an associated burden of 754.000 disability‐adjusted life years (DALYs) [3, 4]. These infections are common in areas with poor sanitation and poverty, mainly in tropical and subtropical regions [5, 6]. The transmission occurs through ingestion of eggs containing L3 larvae, which are present in contaminated environments. After egg ingestion, L3 larvae hatch in the gastrointestinal tract, penetrate the intestinal mucosa, and migrate through various tissues (bloodstream, liver, and lungs). They are subsequently swallowed again, completing their development into adult worms in the small intestine [7]. The full life cycle in humans, from egg ingestion to the development of adult worms, can take up to 76 days [8]. The prolonged migratory phase highlights the public health significance of ascariasis, particularly in terms of liver and lung morbidity [7, 9]. This migratory behavior, along with the release of parasite antigens, elicits a robust local and systemic inflammatory response during the larvae's passage through host tissues [2, 10, 11].

This immune response induces a range of pathological effects in host tissues [12, 13, 14]. However, the majority of research on ascariasis‐related pathology has predominantly examined pulmonary injury, while hepatic involvement has received comparatively less attention [9, 11, 15]. Nevertheless, there are reports describing liver alterations associated with *Ascaris* spp. infection, including liver abscess [16], hepatobiliary disease [17, 18], and pyogenic cholangitis [19]. Notably, ascariasis is a major contributor to hepatobiliary and pancreatic diseases in tropical and subtropical regions where the infection is endemic [20]. However, the mechanisms underlying liver inflammation induced by this parasite remain insufficiently characterized, and the extent of hepatic damage in humans may be significantly underestimated [21]. This knowledge gap is partly attributable to the clinically asymptomatic nature of the hepatic larval stage [22], as most reported liver complications are attributed to adult worm obstruction of bile ducts.

Previous studies have shown that *Ascaris* larval migration through the lungs can trigger prolonged inflammation lasting as long as 9 months post‐infection, leading to chronic pulmonary disease [13] and persistent fibrosis [11]. In contrast, the long‐term hepatic consequences of *Ascaris* larval migration remain largely unexplored. Experimental models using *A. suum* have traditionally employed a high infection dose of 2500 eggs. However, recent work by [4] demonstrated that a lower dose of 250 eggs more closely reflects natural infection conditions, while eliciting a comparable immune response at the peak of infection. Building on these insights, the present study aims to characterize the pathophysiological and immunological impact of *A. suum* infection in the liver using a murine model. We investigated both low (250 eggs) and high (2500) infection doses, under single and repeated exposure conditions, to evaluate liver pathology, biochemical alterations, and inflammatory response during chronic infection, long after the larval migration through the liver.

## 2 Methods

### 2.1 Experimental Design

Six‐week‐old female BALB/c mice were obtained from the Central Animal Facility of the UFMG, Brazil and maintained at the Animal Facility of the Department of Parasitology of the UFMG under controlled temperature conditions (24°C ± 1°C), lighting (12‐h light–dark cycle), and access to filtered water and commercial chow (Nuvilab Cr‐1, Nuvital Nutrients, Brazil) *ad libitum*.

**FIGURE 1:**
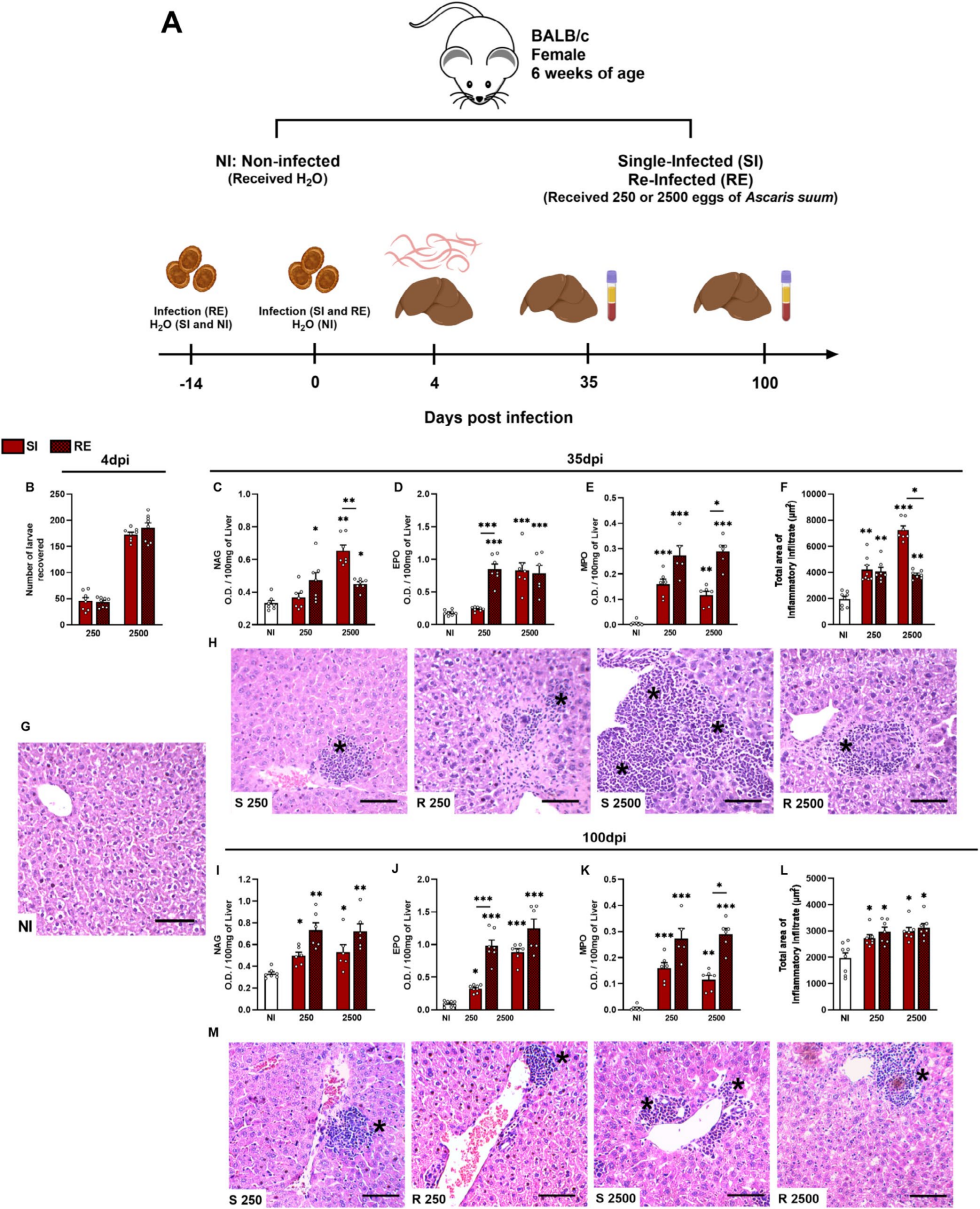
Experimental design, hepatic larval recovery, and inflammatory responses following *A. suum* infection in mice at 35 and 100 dpi. (A) Experimental design. (B) Quantification of liver larvae at 4 days post‐infection (dpi). Hepatic inflammatory markers at 35 dpi: (C) Macrophage‐derived N‐acetilglucosaminidase activity (NAG). (D) Eosinophil‐derived peroxidase activity (EPO), and (E) Neutrophil‐derived myeloperoxidase activity (MPO). (F) Quantitative analysis of inflammatory infiltrate area in the liver parenchyma. (G) Representative H&E‐stained liver sections from non‐infected (NI) animals. (H) Representative H&E‐stained liver sections from infected mice at 35 dpi. Hepatic inflammatory markers at 100 dpi: (I) NAG, (J) EPO, and (K) MPO. (L) Inflammatory infiltrate area at 100 dpi. (M) Representative H&E‐stained sections at 100 dpi. S = single infection. *R* = reinfection. Asterisks (*) indicate inflammatory foci. Data are represented as mean ± Standard Error of the Mean (SEM). For statistical analyses, the Student's *t*‐test was used to evaluate differences between the SI and RE groups at each dose in B. The one‐way ANOVA with Tukey's post hoc test comparing infected groups to NI controls and the respective doses at 35 dpi. Significant differences (*p* ≤ 0.05) are represented by *p < 0.05, **p < 0.01 and ***p < 0.001 (*n* = 8 per group). Asterisks without connecting bars indicate comparisons to the NI group; asterisks with connecting bars denote intergroup comparisons. Non‐significant data were omitted. Scale bars: 200 μm (H&E images).

Mice were randomized in five experimental groups: non‐infected animals (NI), animals single‐infected (SI) with 250 eggs (SI250) or 2500 eggs (SI2500), and animals reinfected (RE) with 250 eggs (RE250) or 2500 (RE2500). Reinfected (RE) animals were infected twice with embryonated *Ascaris suum* eggs at a 14‐day interval, with the first infection administered at day −14 and the second at day 0. In contrast, single‐infection (SI) animals received filtered water by oral gavage at day −14 and a single dose of eggs at day 0. All subsequent time points were calculated relative to day 0. On 4, 35, and 100 days post‐infection (dpi), mice were euthanized via intraperitoneal injection of xylazine/ketamine (8.5 mg/kg and 130 mg/kg) to assess parasite burden (4 dpi) and chronic inflammation (35 and 100 dpi). Following euthanasia, liver tissue and peripheral blood samples were collected for subsequent analysis (Figure 1A).

### 2.2 Parasites and Infection

Adult *A. suum* worms were collected from infected pig intestines discarded by a slaughterhouse (Belo Horizonte, Minas Gerais, Brazil). The worms were kept in saline 0.9% and taken to the Laboratory of Immunobiology and Control of Parasites at the UFMG—Brazil for processing. To obtain eggs, female worms were dissected, and their uteri were mechanically macerated. The resulting material was filtered through 100 μm strainers, resuspended in 0.2 M H_2_SO_4_ at a concentration of 50 mL at 25 eggs/μL, and incubated in a Biological Oxygen Demand (BOD) chamber at 26°C for 100 days to allow complete embryonation, as previously described by [23] and [15]. For infection, the fully embryonated eggs were incubated with 5% (v/v) sodium hypochlorite solution at 37°C in a humidified atmosphere of 5% CO_2_ for 2 h, centrifuged (800 × g, 10 min) and washed with filtered water three times. Then, mice received either 250 or 2500 embryonated eggs in 0.2 mL suspension administered by oral gavage, followed by 0.1 mL sterile water to flush the gavage needle, as described by [15].

### 2.3 Parasitological Analysis in Liver

Parasite burden was assessed by counting the total number of larvae recovered from the liver at 4 dpi. The tissue was collected, sliced with scissors, and placed in a Baermann apparatus for 4 h in phosphate‐buffered saline (PBS) at 37°C. The larvae recovered were fixed (10% formalin) and counted under an optical microscope.

### 2.4 Evaluation of Liver Enzymes

Peripheral blood samples (~600 μL) were collected by cardiac puncture and placed in tubes with EDTA anticoagulant, centrifuged at 5000 × g for 10 min. The plasma was collected to evaluate liver enzymes. Measurements included alanine aminotransferase (ALT, K049), aspartate aminotransferase (AST, K034), alkaline phosphatase (ALP, K021), gamma glutamyltransferase (GGT, K080), total bilirubin (TB, K005) and fractions (direct—DB and indirect—IB), and albumin (K040) using commercial kits (Bioclin Quibasa, Belo Horizonte, Brazil). All assays were conducted following manufacturer instructions with proportional adjustments of reagent volumes based on sample quantity. Sample volumes used were: ALT (10 μL), AST (20 μL), ALP (2 μL), GGT (10 μL), TB and DB (15 μL each) and albumin (1 μL). IB was calculated using the formula IB = TB‐DB. Hemolyzed samples were excluded from the assays.

### 2.5 Quantification of Cytokine

Plasma samples were also used to quantify the levels of IL‐2, IL‐4, IL‐6, IL‐10, IL‐17A, IFN‐γ, and TNF using Cytometry Bead Array (CBA) (Th1/Th2/Th17 BD Biosciences, USA) according to the manufacturer's instructions. The data were acquired using an LSRFortessa flow cytometer (BD Biosciences, USA), and the results were analyzed in FlowJo software (Tree Star, Ashland, OR). IL‐5, IL‐13, and TGF‐β were measured by sandwich ELISA kit (R&D Systems, USA) according to the manufacturer's instructions. The absorbance of the samples was determined in a VersaMax ELISA microplate reader (Molecular Devices, USA) at a wavelength of 492 nm, and the cytokine concentration was calculated by interpolation using a standard curve fitted with five parameters of logistic (5‐PL).

### 2.6 Evaluation of Eosinophil, Neutrophil and Macrophages Activity

Hepatic inflammatory cell activity was evaluated through quantification of cell‐specific enzymatic markers in liver homogenates by the concentration of eosinophil peroxidase (EPO), neutrophil myeloperoxidase (MPO), and N‐acetylglucosaminidase (NAG), respectively, as previously described [10]. After processing, absorbance was determined by a VersaMax ELISA Microplate Reader (Molecular Devices, USA).

### 2.7 Histopathological and Morphometric Analysis

The right hepatic lobe was collected from mice in each group to assess histological changes. The liver was fixed in 10% formalin solution for 2 days, gradually dehydrated in ethanol, diaphanized in xylol, and embedded in paraffin blocks. The blocks were sectioned in a 4 μm thickness, fixed on microscopy slides, and stained with hematoxylin and eosin (H&E) to evaluate tissue damage. Masson's trichrome was also used to assess the area of fibrosis. To assess the severity of liver injury, the slides were examined by two blinded pathologists to the experimental groups. The evaluation was based on a previously validated liver pathology score that categorizes liver parenchymal damage into four grades (Table S1) [24].

The inflammatory infiltrate was quantitatively assessed by enumerating nuclei in histological sections using digital image analysis. For each animal, ten randomly selected fields were captured at 20× magnification using a Motic 2.0 digital imaging system (Motic China Group, Hong Kong). Nuclei counts were performed according to the methodology established by [25], with automated quantification conducted using Image Pro Plus software (v6.0, Media Cybernetics) and expressed in total area in μm^2^. To estimate the fibrosis area, 10 randomly selected fields were captured at 20× magnification and measured according to the total area stained by Masson's trichrome as previously described [26, 27]. The images were obtained using a digital image capture system (Motic 2.0, Hong Kong, China).

### 2.8 Sample Collection, Total RNA Extraction and cDNA Synthesis

The liver was collected, placed in RNA later solution and stored at −80°C until processing. Total RNA was extracted from 30 mg of hepatic tissue using the NucleoSpin RNA Plus Kit (Macherey‐Nagel, Düren, Germany) following the manufacturer's protocol. RNA quality control was performed as previously described by [28]. cDNA was reverse transcribed from 2 μg of total RNA using High‐Capacity cDNA Reverse Transcriptase Kit (Applied Biosystems Inc., Foster City, CA) in a total volume of 20 μL following the manufacturer's instructions. The resulting cDNA products were diluted 1:10 in nuclease‐free water and stored at −20°C until quantitative PCR analysis.

### 2.9 Primers Design and Validation

Gene‐specific primers were designed using mouse nucleotide sequences obtained from NCBI (accession numbers provided in Table 1) and performed using Primer3 web software (v4.1.0; https://primer3.ut.ee/) following the parameters established by [28]. Sequences of Glyceraldehyde‐3‐Phosphate Dehydrogenase (GAPDH), 18S rRNA, β‐actin (ACTB), hypoxanthine phosphoribosyltransferase 1 (HPRT1) and β‐2‐microglobulin (B2M), and TNF were obtained from [28, 29]. The amplicon sequences were aligned with those cataloged in the international databases through the Basic Local Alignment Search Tool (BLAST) to confirm specificity. All details regarding the forward and reverse primers, including sequences, are presented in Table 1. To ensure robust data normalization, a comprehensive validation using five candidate reference genes (GAPDH, 18S, ACTB, HPRT1, and B2M) was conducted. For this step, eight‐week‐old female C57BL/6J and BALB/c mice were randomized and infected with 2500 fully embryonated *A. suum* eggs, while non‐infected animals served as controls. At 4 days post‐infection (dpi), which are considered the peak of larval migration in the liver [15], mice were euthanized via intraperitoneal injection of xylazine/ketamine (8.5 mg/kg and 130 mg/kg) for liver collection and subsequent molecular analysis (Figure S1). This experimental design for reference gene validation followed the same methodology described by Souza and colleagues [28]. This step was essential to identify the most stable reference gene in the liver for *Ascaris* infection studies in BALB/c mice, as previously done in the C57BL/6 model by [28] (Figures S1–S3).

**TABLE 1:** Identification of primers, primer sequences used for relative gene quantification (RT‐qPCR), amplicon size, and accession number of cytokine genes. F: Forward; R: Reverse.

Gene	Primer sequence	Amplicon size (bp)	NCBI accession number
GAPDH	F: ACCCAGAAGACTGTGGATGG R: CACATTGGGGGTAGGAACAC	171	[28]
18S	F: GCCGTTCTTAGTTGGTGGAG R: AACGCCACTTGTCCCTCTAA	129	[28]
ACTB	F: TGTTACCAACTGGGACGACA R: GGGGTGTTGAAGGTCTCAAA	165	[28]
HPRT1	F: TTGGGCTTACCTCACTGCTT R: CTAATCACGACGCTGGGACT	125	[28]
B2M	F: TCTCACTGACCGGCCTGTAT R: GTATGTTCGGCTTCCCATTC	90	[28]
IL‐12/p40	F: TCTTCTGCTTGGTTGGCTTT R: CTCTGCGGGCATTTAACATT	90	NM_001303244.1
IFN‐γ	F: GCGTCATTGAATCACACCTG R: TGAGCTCATTGAATGCTTGG	129	NM_008337.4
TNF	F: TATGGCTCAGGGTCCAACTC R: CTCCCTTTGCAGAACTCAGG	174	[29]
IL‐1‐β	F: CAGGCAGGCAGTATCACTCA R: AGGTGCTCATGTCCTCATCC	95	NM_008361.4
IL‐6	F: CCGGAGAGGAGACTTCACAG R: TCCACGATTTCCCAGAGAAC	102	NM_031168.2
IL‐4	F: CCAAGGTGCTTCGCATATTT R: ATCGAAAAGCCCGAAAGAGT	105	NM_021283.2
IL‐5	F: CACCAGCTATGCATTGGAGA R: TCCTCGCCACACTTCTCTTT	146	NM_010558.1
IL‐17	F: TTCAGGGTCGAGAAGATGCT R: AAACGTGGGGGTTTCTTAGG	112	NM_010552.3
IL‐10	F: CCAGGGAGATCCTTTGATGA R: AACTGGCCACAGTTTTCAGG	96	NM_010548.2
TGF‐β	F: GACTCTCCACCTGCAAGACC R: GACTGGCGAGCCTTAGTTTG	99	NM_011577.2

While B2M had been previously established as the most stable reference gene in liver *Ascaris*‐infected C57BL/6 mice [28], we evaluated its suitability for BALB/c mice in our experimental system. Our analysis revealed that although cycle threshold (Ct) values differed between mouse strains (Figure S2), B2M demonstrated stability across both genetic backgrounds and all infection conditions (Figure S3). This finding was consistently confirmed by different computational algorithms, including RefFinder, using the analytical framework established by [28] (Figures S2 and S3). Consequently, B2M was selected for normalization of gene expression data (Figure S3). Primer performance was rigorously validated and conducted according to established molecular standards. Amplification efficiency (E%) was calculated using the equation E% = [10^(−1/slope)^‐1] × 100 as described by [30]. The range of 90%–115% for the E value was considered ideal for proper function [28], the results are available in Table S2. The specificity of the amplified products was confirmed by visualization of the expected amplicon size on 2% agarose gel electrophoresis (Figure S4). Detailed parameters, including slope, coefficient of determination (R^2^) and melting temperature of each gene are available in Table S2.

### 2.10 RT‐qPCR

The qPCR was performed using a 7500 Real‐Time PCR system (Applied Biosystems) according to the manufacturer's instructions with the PowerUP Sybr green master mix kit (Applied Biosystems). A total volume of 10 μL reactions consisting of 5 μL from the kit, 2 μL cDNA, 0.5 μL of each specific forward and reverse primer (10 μM), and 2 μL of ultrapure nuclease‐free water were used. Triplicates of each sample and a non‐template reaction (negative control) were included. Thermal cycling was performed, starting with an initial step at 50°C for 2 min, followed by 95°C for 10 min, 40 cycles of denaturation at 95°C for 15 s, and annealing/extension at 60°C for 60 s. Melting curve analysis was performed from 70°C to 95°C at 0.1°C/s for all genes assessed. Threshold cycle (Ct) values for each sample were obtained by calculating the arithmetic mean of triplicate values.

### 2.11 Statistical Analysis

Grubb's test was used to detect possible sample outliers and Shapiro–Wilk normality test was performed to verify data distribution. For comparisons between two groups, a *t*‐test with Welch's correction was applied, while evaluation between three or more groups was performed using Analysis of Variance (ANOVA) or Kruskal‐Wallis test, followed by Tukey's or Dunn's post‐test, respectively, depending on the data distribution. GraphPad Prism 9.3.0 (GraphPad Software Inc., USA) was used for statistical analysis. To create the heatmaps, we used the online tool ClustVis 2.0 [31]. We performed the principal component analysis in R (version 4.3.1) using the FactoMineR and factoextra packages to visualize group clustering, while ggplot2 was used to generate the 95% confidence ellipses for each experimental group. All tests were considered statistically significant at *p* ≤ 0.05.

## 3 Results

### 3.1 Infection With *A. suum* Elicits Persistent Hepatic Inflammation Even at Lower Doses, and Reinfection Does Not Confer Protection Against Parasite Burden, Regardless of Infection Dose

To assess early parasite migration, we quantified the number of larvae in the liver at 4 dpi in single infected (SI) and reinfected (RE) mice. In the SI group, mice infected with 2500 eggs had a mean of 172 larvae in the liver (6.91% of the inoculum), while those infected with 250 eggs had fewer larvae, with a mean of 45 (18.3%) (Figure 1B and Table S3). In contrast, after reinfection, no differences were observed among groups: 185 in RE2500 and 43 in RE250, corresponding to 7.43% and 17.45% of larvae recovered, respectively (Figure 1B and Table S3).

At 35 dpi, we assessed hepatic inflammatory infiltrates caused by larval migration. N‐acetylglucosaminidase (NAG) activity showed significant elevation in both reinfected groups (RE250 and RE2500) compared to NI control. However, the SI2500 group exhibited the highest NAG activity, differing significantly from both NI and RE2500 (Figure 1C). Eosinophil peroxidase (EPO) activity was elevated in RE250, SI2500, and RE2500 groups, indicative of ongoing eosinophilic inflammation (Figure 1D). Myeloperoxidase (MPO) activity was altered across all infected groups; specifically, it was significantly higher in both reinfected groups (RE250 and RE2500) compared to NI, while remaining lower in SI groups (Figure 1E). Histopathological analysis revealed similar infiltrate intensity in mice infected with 250 eggs, regardless of exposure history, as well as in the RE2500 (Figure 1F). Hepatic infiltrates were characterized as mixed, comprising neutrophils and eosinophils, but composed of predominantly mononuclear cell types. Representative histopathological images are presented in Figure 1G,H.

Given the absence of detectable larvae in the liver beyond 10 dpi or systemically beyond 14 dpi [15], we extended our assessment to 100 dpi to assess long‐term hepatic inflammation. Inflammatory infiltrates persisted in all infected groups at this chronic phase (Figure 1I–M). Enzymatic assays confirmed ongoing inflammation, demonstrating significantly increased NAG activity in RE groups (Figure 1I), and elevated EPO activity in RE250, SI2500, and RE2500 groups, whereas levels were reduced in SI250 (Figure 1J). MPO activity was likewise elevated in all infected groups, most notably in RE groups (Figure 1K). Quantification of inflammatory infiltrate areas revealed comparable elevation across all infected groups, regardless of inoculum dose or exposure frequency (Figure 1L), with infiltrates predominantly composed of mononuclear cells. Representative histopathological images are shown in Figure 1M.

### 3.2 Persistent Inflammation Induced by *A. suum* Infection Leads Progressive Hepatic Injury and the Development of Cholestastic Disease

**FIGURE 2:**
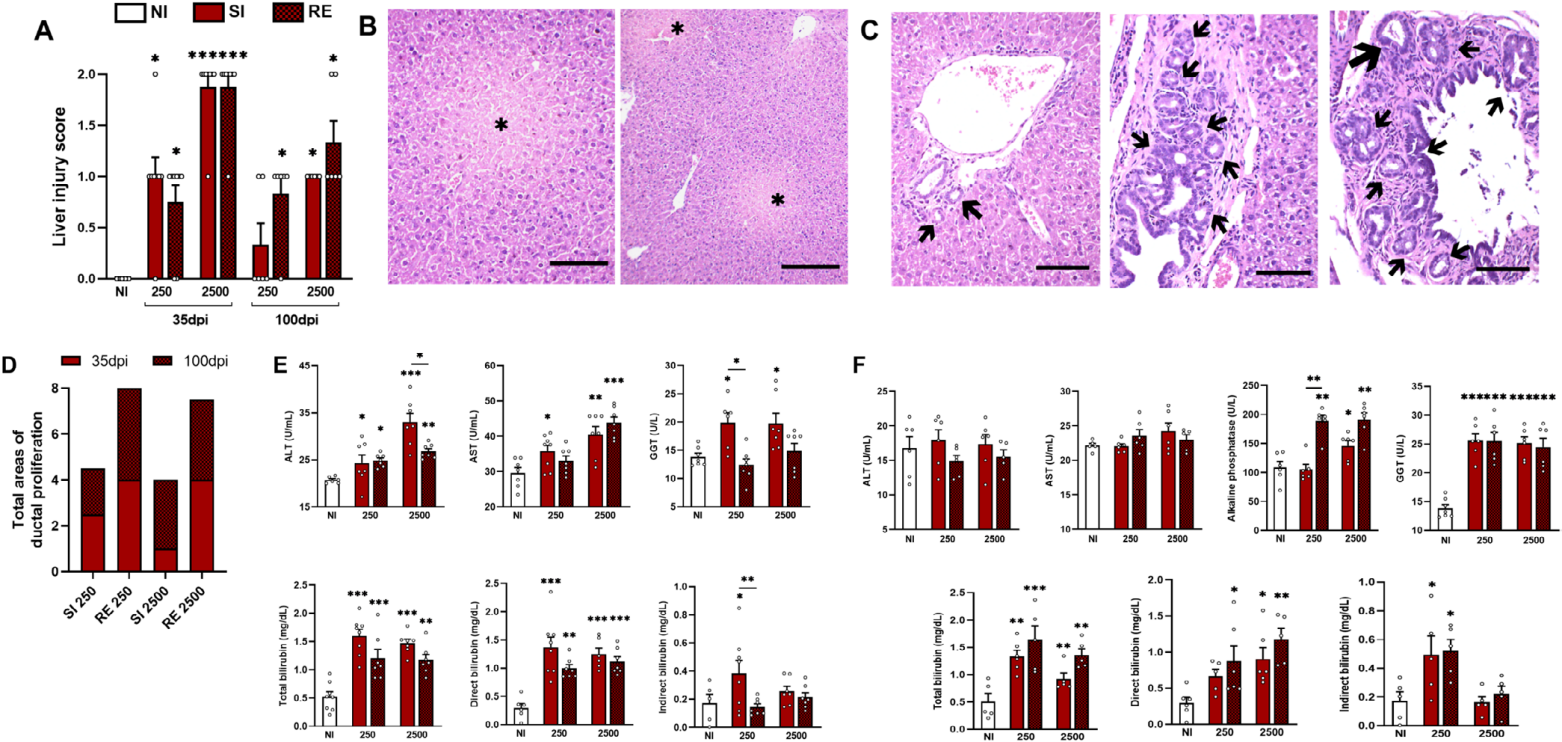
Liver injury at 35 and 100 dpi in mice single and reinfected with different doses of *A. suum*. (A) Liver histopathology scores at 35 and 100 dpi. (B) Representative H&E‐stained liver sections depicting necrotic areas. (C) Representative H&E‐stained liver sections highlighting regions of ductal proliferation with varying intensity. (D) Quantification of ductal proliferation areas in mice infected at 35 and 100 dpi. (E) Plasma levels of Alanine Aminotransferase (ALT), Aspartate Aminotransferase (AST), Gamma‐Glutamyltranspeptidase (GGT), Total Bilirubin (TB), and direct (DB) and indirect (IB) fractions at 35 dpi. (F) Plasma levels of Alkaline Phosphatase (ALP), Gamma‐Glutamyltranspeptidase (GGT), Total Bilirubin (TB), and direct (DB) and indirect (IB) fractions at 100 dpi. Data are represented as mean ± SEM. NI = Non‐infected groups. SI = single infection. RE = reinfection. For statistical analyses, one‐way ANOVA followed by Tukey's test was used to evaluate differences between NI groups and respective doses. Significant differences (*p* ≤ 0.05) are represented by *p < 0.05, **p < 0.01 and ***p < 0.001 (*n* = 8 per group). Asterisks without connecting bars indicate comparisons to the NI group; asterisks with connecting bars denote intergroup comparisons. Non‐significant data were omitted. Scale bars: 200 μm (H&E images).

Liver injury scores were assessed at 35 dpi in all infected groups, with the highest scores observed in both SI and RE infected groups receiving 2500 eggs (Figure 2A). At 100 dpi, scores remained elevated in SI250 and 2500 groups, along with the RE 2500 group (Figure 2A). Histopathological analysis identified areas of hepatic necrosis, most prominently in mice receiving higher doses at 35 dpi (Figure 2B). Although the severity of necrosis was reduced at 100 dpi, lesions remained detectable in animals from higher‐dose groups and the RE250 group. Notably, histological evaluation also identified areas of ductal proliferation, which can be seen in cholestatic disease (Figure 2C). These alterations were present in all groups, regardless of dose or exposure history, at both 35 and 100 dpi. In some cases, proliferation was mild, characterized by ducts displaying slight dilation or normal caliber, accompanied by discrete epithelial hyperplasia and periductal inflammatory infiltrates. In other cases, moderate ductal proliferation was evident, frequently associated with focal fibrosis, as confirmed by Masson's trichrome staining. However, the distribution and severity of ductal alterations lacked a consistent pattern across infected animals, suggesting these changes are not dose‐dependent. A notable finding from quantitative analysis was that animals in SI groups exhibited fewer areas of ductal proliferation than those in RE groups (Figure 2D). Furthermore, the SI2500 group showed increased proliferative areas at 100 dpi relative to 35 dpi (Figure 2D). By 100 dpi, partial restoration of liver architecture was observed in some animals; however, hyperplastic and dilated bile ducts persisted in multiple samples. Importantly, no histological evidence of intrahepatic cholestasis, bile duct epithelial injury, or bilirubin deposition was observed at either 35 or 100 dpi time points.

Plasma biochemical analyses revealed significantly increased ALT levels in all infected groups at 35 dpi, notably in the SI2500, whereas AST levels were increased in all groups infected with 2500 eggs (Figure 2E). At 100 dpi, however, both ALT and AST levels decreased across all groups, returning to control levels (data not shown). Significant alterations in ALP were not observed at 35 dpi, but were elevated at 100 dpi in the RE250, SI2500, and RE2500 groups (Figure 2F). GGT levels were elevated at 35 dpi in all SI groups, regardless of dose, and increased in all groups at 100 dpi (Figure 2E,F). Total bilirubin was elevated at 35 dpi, with direct bilirubin increased in all groups and indirect bilirubin elevated only in SI250 (Figure 2E). At 100 dpi, total bilirubin remained elevated in all groups, with direct bilirubin persistently increased in all groups, whereas indirect bilirubin was elevated in the 250‐egg groups (Figure 2F). Albumin levels demonstrated no alterations at either 35 or 100 dpi in any group (data not shown).

### 3.3 Chronic Inflammation Caused by *A. suum* Leads to the Emergence of Fibrosis That Persists in the Tissue

**FIGURE 3:**
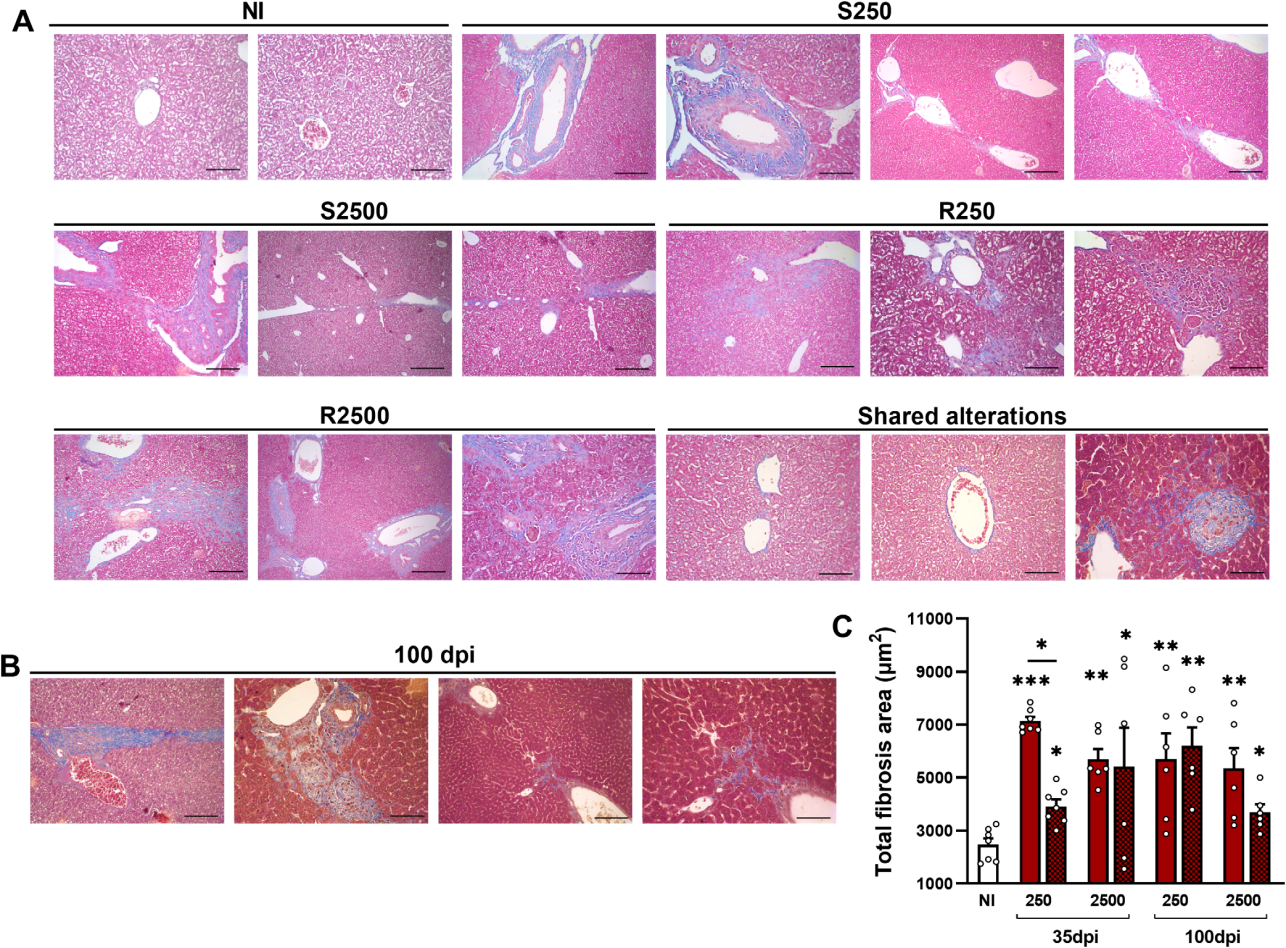
Areas of fibrosis in the liver at 35 and 100 dpi of mice single and reinfected with different doses of *A. suum* and frequency of exposures. (A) Representative areas of fibrosis in the liver at 35 dpi; and at (B) 100 dpi according to the number of exposures stained with Masson's Trichrome. (C) Morphometric quantification of fibrotic areas at 35 and 100 dpi. NI = Non‐infected animals. S = Single‐infected mice and *R* = Reinfected mice. Data represent mean ± SEM. Statistical analysis at 100 dpi was performed using one‐way ANOVA with Tukey's post hoc test. Significant differences (*p* ≤ 0.05) versus the NI control are denoted as follows: *p < 0.05, **p < 0.01 and ***p < 0.001 (*n* = 8 per group). Asterisks without connecting bars indicate comparisons to the NI group; asterisks with connecting bars denote intergroup comparisons. Non‐significant data were omitted. Scale bars: 200 μm (H&E images).

To better characterize the extent and dynamics of fibrosis, Masson's trichrome staining was performed. Histopathological analysis revealed extensive fibrotic remodeling in the liver at both 35 and 100 dpi. All infected groups displayed periportal fibrosis and centrilobular involvement at both time points. At 35 dpi, SI250 animals exhibited mild to moderate periportal fibrosis, with bridging fibrosis observed in three cases. RE250 animals showed a similar pattern but with increased parenchymal extension. In the SI2500 group, fibrosis was more pronounced, with clearly defined septa connecting portal tracts. This phenotype was further exacerbated in RE2500 animals, which showed abundant septa formation and advanced lobular remodeling (Figure 3A). At 100 dpi, the SI250 and RE250 groups retained the fibrotic pattern observed at 35 dpi, with no substantial progression. In contrast, SI2500 and RE2500 animals displayed increased septa formation and granulomatous inflammation (Figure 3B), particularly marked in the RE2500 group. Together, these findings demonstrate that both parasite burden and repeated exposure contribute to the persistence and severity of hepatic fibrosis following *A. suum* infection.

Analysis of the area of fibrosis revealed that reinfected animals exhibited a trend toward smaller fibrotic areas compared to their respective single‐infection (SI) groups (Figure 3C). Notably, the SI250 group showed a trend toward the largest fibrotic areas, suggesting that lower parasite burdens during early infection may induce more intense focal hepatic damage. By 100 dpi, fibrotic lesions persisted across all infected groups, with variable intensity among individual animals (Figure 3B,C), underscoring the chronic and heterogeneous nature of liver injury induced by *A. suum*.

### 3.4 Transcriptional Reprogramming of Inflammatory Genes Occurs Post‐*Ascaris suum* Infection, Exhibiting Time‐Dependent Heterogeneity

**FIGURE 4:**
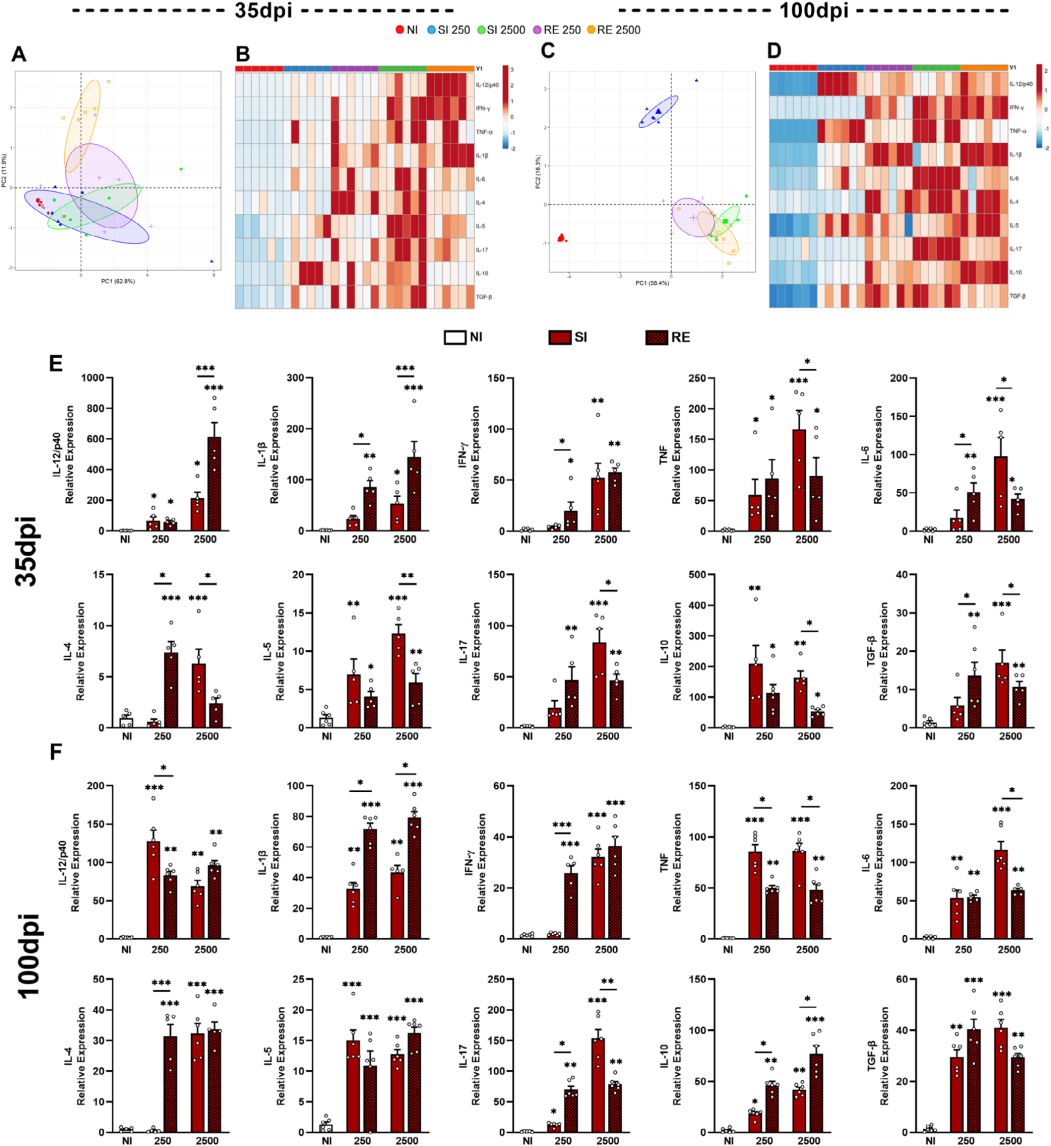
Dynamic of expression of selected inflammatory genes in liver after *Ascaris suum* infection in mice 35 and 100 dpi. (A) Principal Component Analysis (PCA) of 10 cytokine transcripts at 35 dpi from liver of mice infected with *A. suum* at different doses and number of exposures. (B) Heatmap of cytokine expression profiles at 35 dpi from liver of mice infected with *A. suum*. (C) Principal Component Analysis (PCA) of 10 cytokine transcripts at 100 dpi from liver of mice infected with *A. suum* at different doses and number of exposures. (D) Heatmap of cytokine expression profiles at 100 dpi from liver of mice infected with *A. suum*. (E) Relative expression of specific cytokines at 35 dpi. (F) Relative expression of specific cytokines at 100 dpi. Data presented as mean ± Standard Error of the Mean (S.E.M). NI = Non‐infected animals. SI = single infection. RE = reinfection. For statistical analyses, the ANOVA test with Tukey post‐test was used to evaluate differences between the NI groups and the respective doses at 100 dpi. Significant differences (*p* ≤ 0.05) are represented by *p < 0.05, **p < 0.01 and ***p < 0.001 (*n* = 6 per group). Asterisks without connecting bars indicate comparisons to the NI group; asterisks with connecting bars denote intergroup comparisons. Non‐significant data were omitted.

Analysis of cytokine gene expression at 35 and 100 dpi revealed upregulation in most genes across infected groups, regardless of infection dose or exposure frequency. To characterize the dynamics of this response, we performed a principal component analysis (PCA) at both time points. At 35 dpi, PCA demonstrated similar gene expression patterns across infected groups, but only the RE2500 group exhibited slight separation from the others, suggesting highest immunomodulation (Figure 4A). Overall, infected groups displayed homogeneous transcriptional profiles, corroborated by heatmap analysis, indicating a convergent immune activation signature driven by *A. suum* infection, particularly pronounced in the RE2500 group (Figure 4B). At 100 dpi, the PCA revealed that the SI2500, RE250, and RE2500 groups maintained similar transcriptional profiles, characterized by markedly elevated expression of multiple cytokine genes, suggesting protracted immune activation. In contrast, the SI250 group manifested a distinct expression profile, forming a discrete cluster in PCA space (Figure 4C). This divergence implies potential dose‐dependent immune modulation during chronic infection. These observations align with the heatmap analysis (Figure 4D), which confirms the clustering of gene expression profiles among most infected groups and the segregation of SI250. At 35 dpi, RE2500 exhibited significantly heightened IL‐12/p40 and IL‐1β expression versus all groups. SI2500 displayed elevated TNF, IL‐5, IL‐6, and IL‐17 expressions. Both RE and SI groups receiving 2500 eggs exhibited heightened IFN‐γ expression. The RE250 group demonstrated IL‐17 levels equivalent to RE2500, alongside increased expression of IL‐10, TGF‐β, and IL‐4, comparable to high‐dose groups. The SI250 group exhibited exclusively elevated TNF, IL‐5, and IL‐10 expression (Figure 4E).

At 100 dpi, transcriptional elevation persisted for most cytokines. IFN‐γ and IL‐4 expression demonstrated analogous profiles between the RE250 and SI2500/RE2500 groups. TNF and IL‐1β expression patterns diverged: TNF increased in SI groups, whereas IL‐1β was elevated in RE groups irrespective of dose. The SI250 group exhibited maximal IL‐12 expression relative to all other groups. IL‐5 and TGF‐β levels remained comparable across all cohorts. Peak IL‐6 and IL‐17 expression occurred in the SI2500 group. Both RE250 and RE2500 groups displayed similar upregulation of IL‐12, IFN‐γ, TNF, IL‐1β, IL‐6, IL‐4, IL‐5, and IL‐17. Collectively, these results demonstrate a heterogeneous cytokine milieu during chronic inflammation of *A. suum* infection (100 dpi), indicative of sustained immunomodulation that remodels the hepatic microenvironment (Figure 4F).

### 3.5 *Ascaris suum*‐Induced Hepatic Injury Promotes a Mixed Th1/Th2 Polarized Systemic Immune Response Associated With Infectious Dose and Exposure Frequency

**FIGURE 5:**
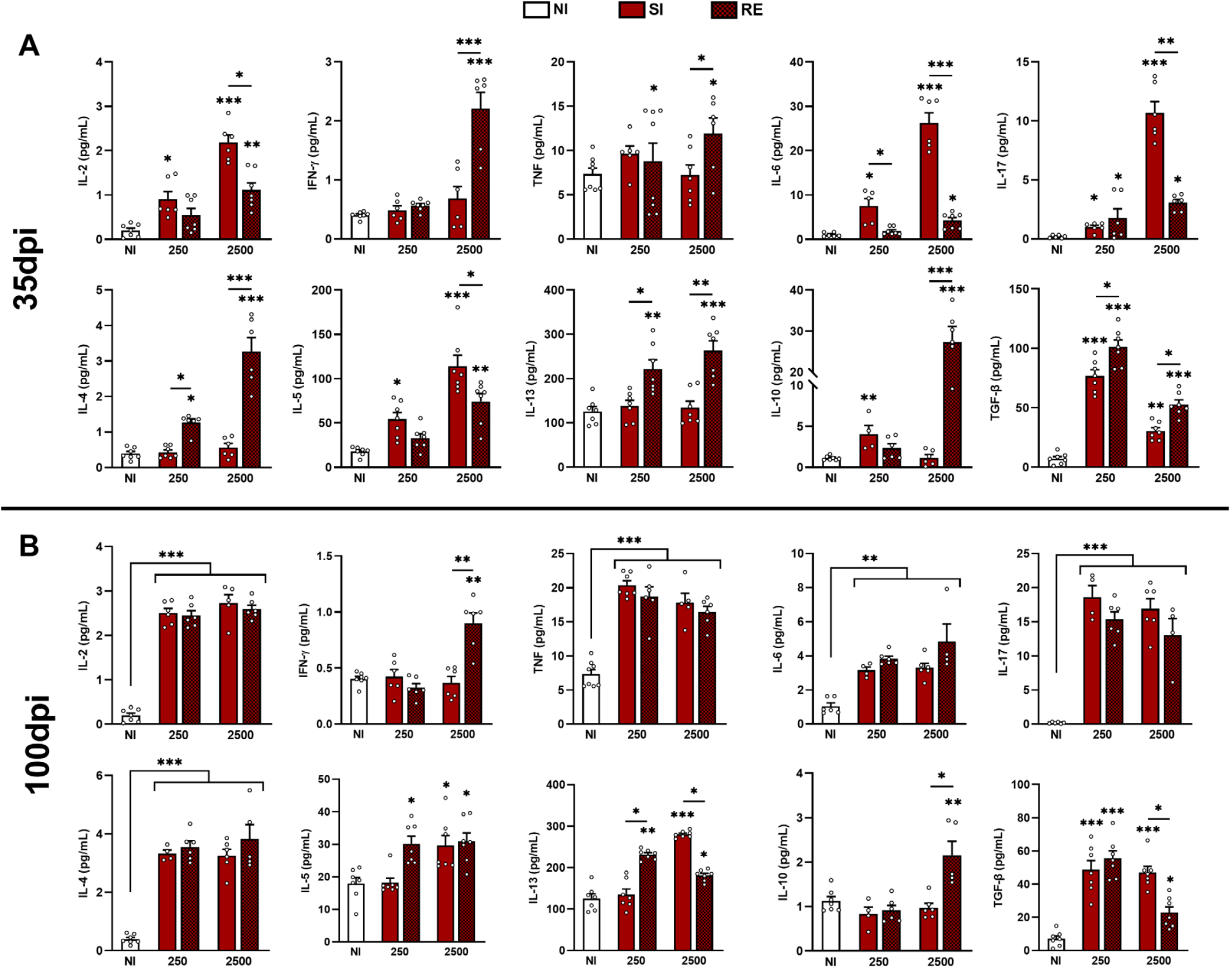
Systemic cytokine levels following single or reinfection with *Ascaris suum* in mice at varying doses, evaluated at 35 and 100 dpi. (A) Systemic cytokine profile at 35 dpi and (B) 100 dpi quantified by CBA and ELISA. Data are expressed as mean ± Standard Error of the Mean SEM. NI = Non‐infected animals. SI = single infection. RE = reinfection. Statistical analysis was conducted using one‐way ANOVA with Tukey's post hoc test to assess differences between NI controls and infected groups at corresponding doses/timepoints. Significant differences (*p* ≤ 0.05) are represented by *p < 0.05, **p < 0.01 and ***p < 0.001. Asterisks without connecting bars indicate comparisons to the NI group; asterisks with connecting bars denote intergroup comparisons. Non‐significant data were omitted.

Based on the experimental findings, we investigated systemic cytokine profile alterations (IL‐2, IFN‐γ, TNF, IL‐4, IL‐5, IL‐6, IL‐10, IL‐13, IL‐17A, and TGF‐β) at 35 dpi. Our analysis revealed distinct response patterns across experimental groups. Higher‐dose infection (2500 eggs) elicited elevated systemic cytokine production, particularly in reinfected (RE2500) animals. At 35 dpi, the RE2500 group exhibited increased IFN‐γ, TNF, IL‐4, and IL‐10, whereas IL‐2, IL‐6, and IL‐17A were elevated in the SI250 group (Figure 5). Although cytokine levels did not reach those observed in the high‐dose group, reinfection with 250 eggs led to significant changes in IL‐2, IL‐6, and IL‐10 compared to non‐infected (NI) controls. Notably, IL‐4 and IL‐17A levels were also elevated following reinfection (Figure 5A). These data indicate that while lower infection doses do not provoke robust inflammatory responses, they induce persistent cytokine dysregulation at 35 dpi (Figure 5A). At 100 dpi, systemic increases in IL‐2, TNF, IL‐4, IL‐17, and IL‐6 occurred across all infected groups, irrespective of dose or exposure frequency (Figure 5B). TGF‐β levels were elevated universally but were most pronounced in SI250/RE250 and SI2500 groups, while IL‐13 elevation was restricted to RE250 and SI2500 groups. IL‐10 and IFN‐γ increases occurred exclusively in the RE2500 group (Figure 5B).

## 4 Discussion

This study employed a murine model to investigate the effects of *A. suum* larval migration on hepatic pathology, evaluating the effects of different infection doses and exposure frequencies over extended post‐infection intervals. Previous studies have established that lower infectious doses (250 eggs) more accurately replicate the dynamics of natural infection [4]. To extend these observations, we evaluated liver pathology in both single‐infection and reinfection scenarios, incorporating insights from [10] regarding the clinical relevance of reinfection, a common phenomenon in human populations. Critically, our data demonstrate that ascariasis leads to chronic liver disease characterized by a cholestatic pattern of injury, thereby promoting hepatic fibrosis and progressive functional impairment.

Although rarely emphasized, hepatic injury associated with ascariasis represents a significant contributor to hepatobiliary and pancreatic diseases in endemic regions worldwide [18, 20]. These cases are primarily linked to heavy parasitic burden, where adult worms migrate from the jejunum to the duodenum and penetrate the ampulla of Vater, invading the biliary tract and occasionally extending to the gallbladder and pancreatic duct. Once lodged in these structures, the parasites trigger a broad spectrum of pathophysiological changes [32, 33]. In contrast, a detailed understanding of early hepatic involvement during the larval migratory phase remains limited, as it typically requires post‐mortem examination for assessment [34]. The absence of noticeable clinical symptoms during this phase contributes to its frequent oversight in both clinical practice and research. Nevertheless, hepatic involvement during the larval migratory phase has been well documented in other experimental models, such as pigs [35] and mice [10, 11]. Interestingly, parallel findings have been reported in humans. In a series from the Kashmir Valley, biliary ascariasis accounted for 14.5% of 510 patients admitted with liver abscess, with worms (live or dead) identified within the abscess cavity in a subset of cases, with or without associated *Ascaris* in the bile ducts [36]. These findings closely resemble the lesions identified in animal models, further supporting the translational relevance of our study.

Several studies have suggested the occurrence of “self‐cure” in hosts infected with *Ascaris* spp. [15, 22, 37]. In pigs, for example, maturation into adult worms is frequently impaired by infectious dose, implicating the liver as a potential site of early larval control [22, 38]. Gazzinelli‐Guimarães and colleagues [15] demonstrated in mice that of 2500 ingested eggs, only ~150 larvae reached peak pulmonary infection at 7–8 dpi, while ~400 larvae accumulated in the liver at 4–5 dpi. This evidence reinforces the liver's critical role in mediating larval migration containment and parasite burden limitation.

Prior studies have established that larval migration through the liver induces substantial inflammation and provokes host immune response [11, 14]. Although macroscopic white lesions were absent in our murine model, significant hepatic fibrosis and granuloma formation were observed at both 35 and 100 dpi, confirmed by Masson's trichrome staining. Notably, the hepatic immune response in the RE250 group mirrored that of higher‐dose groups. This parallels findings by [4], who reported similar pulmonary inflammatory responses in both low‐ and high‐dose infections. One possible explanation for this pattern relates to differences in the timing and magnitude of immune activation. Experimental studies show that lower infectious doses tend to elicit weaker and slower immune responses, whereas higher parasite burdens trigger stronger inflammatory activation and faster recruitment of effector cells [4, 10, 15, 39]. In murine models of larval ascariasis, this dose‐dependent immune activation has been associated with earlier restriction of larval migration and faster initiation of tissue repair. Accordingly, delayed immune activation during low‐dose infections may allow larvae to persist longer in host tissues, which is consistent with the higher larval recovery observed in the liver in our low‐dose group (18.3%) compared with the high‐dose group (6.91%). A similar scenario was observed in the lungs using different doses in another study [39]. Following reinfection, inflammatory responses declined significantly by 35 dpi in the higher‐dose groups, converging with the immune profiles observed in low‐dose infections. Consistent with previous reports [10], our data also indicate that the host fails to control hepatic parasite burden upon reinfection, regardless of the initial inoculum.

Furthermore, ductal proliferation was observed in the mice persisting up to 100 dpi—nearly 3 months post‐complete parasite clearance [2, 13]. This indicates that the hepatic damage induced by the parasite persists well beyond the period of active infection. The observed ductal proliferation likely reflects a reactive biliary response to sustained liver damage, a feature commonly seen in chronic liver disease, such as non‐alcoholic fatty liver disease [40], alcohol‐related hepatitis [41], and viral hepatitis, such as hepatitis B and C [42, 43]. Notably, the extent of ductal proliferation directly correlates with patient mortality in these cases [40]. The ductular reaction observed in our model likely reflects an immune‐driven remodeling process within the portal microenvironment. In chronic cholestatic disorders, inflammatory cytokines such as IL‐17 have been implicated in promoting cholangiocyte proliferation and sustaining portal inflammation by acting directly on biliary epithelial cells, enhancing their proliferative and pro‐inflammatory responses while amplifying neutrophil recruitment and local cytokine production [44]. Concurrently, TGF‐β is a central mediator of fibrogenesis and extracellular matrix deposition and may contribute to both portal fibroblast activation and the progression of periportal fibrosis [45].

In our study, the persistent upregulation of pro‐inflammatory and pro‐fibrotic genes at 35 and 100 dpi, together with sustained portal inflammation and collagen deposition, supports a model in which the chronic immune response to larval migration promotes a self‐perpetuating cycle of ductular expansion and fibrogenesis. Although causality cannot be definitively established from the present data, the integrated molecular and histological findings strongly suggest that immune‐mediated signaling pathways contribute directly to the development of the chronic cholestatic phenotype.

This study is the first to report ductal proliferation and extensive fibrosis in histopathological assessments following *Ascaris suum* larval infection, a chronic pathological feature not reported in prior studies focused predominantly on acute infection phases [10, 14, 15]. Critically, ascariasis remains neglected yet highly prevalent in regions co‐endemic for hepatotropic parasites, such as malaria and schistosomiasis [22]. Such co‐infections, particularly with *Plasmodium*, potentiate injury, consistent with worsened outcomes in *Plasmodium*–*Ascaris* co‐infected mice [37]. Similarly, concurrent hepatotropic viral infections (e.g., HBV, HCV) may exacerbate hepatic damage [46]. Furthermore, combining *A. suum* infection with bleomycin‐induced pulmonary fibrosis in a murine model significantly elevated biochemical markers levels and increased hepatic inflammatory foci caused by larval migration, demonstrating that comorbidity exacerbates liver damage, as previously described [11]. Underlying comorbidities (e.g., alcohol misuse, dyslipidemia, obesity) further synergize with ascariasis to amplify injury, promote ductal proliferation, and accelerate fibrogenesis. While our findings underscore significant clinical implications for long‐term hepatic sequelae in co‐endemic settings, targeted investigations are warranted to validate these observations and elucidate cumulative pathological mechanisms.

Experimental models of single infections have established involvement of pro‐inflammatory cytokines such as TNF, IL‐6, and IL‐1β [2, 15]. In contrast, repeated exposures result in a mixed inflammatory profile with prominent Th2/Th17 components [4, 10, 47]. In this sense, reinfection with *A. suum* induced sustained upregulation of pro‐inflammatory cytokine genes (IL‐12/p40, IFN‐γ, TNF, IL‐1β, and IL‐6), persisting through 100 dpi in both low‐ and high‐dose groups. At the same time, we detected significant elevation of IL‐4, IL‐5, and IL‐17, together with elevated IL‐10 and TGF‐β. The rise in IL‐10 and TGF‐β reflects pathways classically linked to tissue repair and wound healing, particularly in the case of TGF‐β. Larval ascariasis is known to provoke intense pulmonary injury during migration, followed by prolonged tissue remodeling and fibrotic changes [2]. In this context, sustained production of IL‐10 and TGF‐β is biologically coherent, as both cytokines can contribute to resolution of inflammation while simultaneously promoting extracellular matrix deposition and structural reorganization. In the present study, we demonstrate that a similar fibrotic remodeling dynamic is established in the liver, supporting the idea that repeated *Ascaris* exposure drives a coordinated inflammatory and pro‐repair response across affected organs.

Comparative analysis of RE250 and SI2500 revealed that reinfection at lower doses triggered a pro‐inflammatory profile comparable to high‐dose primary infection, aligning with larval migration patterns reported in the lungs [4]. Notably, RE2500 exhibited the most pronounced response, demonstrating dose‐dependent amplification of inflammation. At 100 dpi, elevated IL‐4 and IL‐5 expression persisted, consistent with prior studies [2, 13]. Sustained IL‐17 expression further indicated a mixed inflammatory state and implicated this cytokine in fibrosis development, as evidenced for *A. suum* [2] and *Schistosoma japonicum* infection [48]. We posit that IL‐17 synergizes with IL‐4 to drive fibrotic remodeling, supported by mechanistic studies [11, 49, 50]. Critically, persistent inflammation in RE250, despite lower parasite burden, suggests reinfection sustains tissue immunopathology independently of larval load. This parallels histopathological and immune patterns in SI2500 indicating repeated *A. suum* exposure potentiates tissue injury. The exacerbated inflammatory profile in RE2500 implies cumulative damage and heightened risk of chronic sequelae. Collectively, these data establish ascariasis, particularly under reinfection scenarios, as a driver of persistent immunopathology with significant implications for hepatic and pulmonary morbidity.

Systemic cytokine profiling revealed significant alterations in secreted mediators. During reinfection at higher doses (RE2500, 35 dpi), elevated IFN‐γ, IL‐4, and IL‐10 levels were sustained. Conversely, single infection induced more pronounced increases in IL‐6 and IL‐2, particularly at the 2500‐egg dose. TNF was markedly elevated during reinfection and at higher doses. IL‐17 demonstrated peak elevation in SI2500 but increased across all groups. By 100 dpi, IL‐2, TNF, IL‐6, IL‐17, and IL‐4 remained elevated universally, independent of dose or exposure frequency. IFN‐γ persisted exclusively in RE2500, mirrored by IL‐10 (which also showed group‐specific elevation). IL‐5 increased in both RE2500 and SI/RE2500 groups. Notably, TGF‐β was elevated in all cohorts, with minimal expression in RE2500. These patterns align with human ascariasis data [1]. The systemic persistence of these cytokines suggests pluripotent effects beyond hepatic involvement, potentially impacting extrahepatic organs [51]. Collectively, these findings demonstrate that *A. suum* infection drives sustained systemic immunomodulation, detectable at 100 dpi, extending beyond local tissue pathology.

A pivotal translational insight from this study is the demonstration that ascariasis‐associated cholestasis extends beyond mechanical obstruction by adult worms in the biliary tract. Historically, the pathophysiology of *Ascaris*‐induced cholestasis and jaundice has centered on physical ductal obstruction by parasites [16, 17, 18, 19, 20]. However, our experimental data reveal persistent cholestatic injury even in the absence of adult worms at 100 dpi, establishing that larval migration and subsequent inflammation independently drive cholestasis, robustly across doses and exposures. These findings necessitate revision of the prevailing ascariasis paradigm and redefine liver disease pathogenesis. Critically, they underscore the clinical imperative to integrate immunopathogenic mechanisms with mechanical factors in diagnosis and management. Assessing these findings in humans living in endemic areas will be critical. This is particularly relevant for adolescents/adults, frequently excluded from mass drug administration programs due to presumed lower burdens, who may harbor significant inflammatory sequelae. Finally, considering the pattern of liver fibrosis observed in this study, it is also reasonable to hypothesize that differentiating ascariasis from hepatosplenic schistosomiasis will be essential, as both parasites may coexist in endemic regions and potentially contribute to periportal fibrosis, confounding diagnosis and attribution of liver disease.

## 5 Conclusion

Using a novel approach, our data establish for the first time that *Ascaris suum* infection in the mice model drives persistent inflammation through 100 dpi, culminating in hepatic dysfunction, fibrosis, and cholestatic pathology, even at low infectious doses. Although exposure to 250 eggs induces attenuated acute injury relative to higher doses, chronic inflammation and structural alterations persist long term. These outcomes underscore the critical need for enhanced control strategies in endemic regions, where reinfection and hepatotropic co‐infections are prevalent. Notably, *A. suum*‐induced cholestasis may exacerbate pre‐existing hepatopathies, positioning ascariasis as a significant, modifiable risk factor for global liver morbidity. Elucidating the pathogenic immune axes underlying this pathology is imperative for developing targeted therapeutic and prophylactic interventions. Collectively, these findings necessitate reframing ascariasis beyond a neglected parasitic disease toward recognition as a determinant of integrated liver health burdens worldwide.

## Author Contributions

Conceived and designed the experiments: L.L.B, G.G.L.C., J.L.N.S. Performed the experiments: J.L.N.S., C.C.O.A., A.R.A.‐P., F.R.S., E.A.O., I.S.O., I.B.D., A.M.S.E, M.E.C., R.M.M.B., L.M.D.M., Funding acquisition: G.D.C., N.B., G.G.L.C, R.T.F., R.C.R., and L.L.B. Supervision: R.T.F., R.C.R., and L.L.B. Contributed reagents/materials/analysis tool: G.D.C., R.T.F., R.C.R., and L.L.B. Writing original paper draft: J.L.N.S., A.R.A.‐P., L.M.D.M., N.B., G.G.L.C., R.C.R., and L.L.B. All other authors revised the data and discussed the manuscript before sending the final version.

## Funding

Pró‐reitoria de pesquisa of UFMG, Fundação de Amparo à Pesquisa do Estado de Minas Gerais/FAPEMIG, Brazil (Grant # APQ‐02628‐24), Rede Mineira de Imunobiológicos (RED‐00067‐23), and Conselho Nacional de Desenvolvimento Científico e Tecnológico (CNPq) Demanda Universal (Grant # 403278/2023‐6). RTF (CNPq Grant #305514/2022‐9), RCR (CNPq Grant # 313839/2023‐9) and LLB (CNPq #310311/2023‐3) are research fellows of the CNPq and RCR e GDC are supported by a grant from the FAPEMIG (Rede Mineira de Pesquisa Translacional em Imunobiológicos e Biofármacos no Câncer—REMITRIBIC, RED‐00031‐21). NB is supported by the Yale Physician Scientist Development Award and CTSA Grant Number UL1 TR001863 from the National Center for Advancing Translational Science (NCATS), a component of the National Institutes of Health (NIH), Robert E. Leet and Clara Guthrie Patterson Trust Mentored Research Award, Bank of America, Private Bank, Trustee, and Burroughs Wellcome Fund/American Society of Tropical Medicine and Hygiene Tropical Medicine Fellowship. Its contents are solely the responsibility of the authors and do not necessarily represent the official views of the funders. JLNS is supported by a postdoctoral fellowship (Programa Institucional de Pós‐Doutorado—PIPD) from CAPES.

## Ethics Statement

All procedures performed were conducted according to ARRIVE (Animals in Research: Reporting In Vivo Experiments) guidelines and the Brazilian College of Animal Experimentation (COBEA) and approved by the local Animal Ethics Committee (CEUA) of the Universidade Federal de Minas Gerais (UFMG), under protocol number: 56/2018 and 178/2021.

## Conflicts of Interest

The authors declare no conflicts of interest.

## Supporting information


**Figure S1:** Experimental design to evaluation of reference genes in the liver of BALB/c and C57BL/6j at the peak of infection of *A. suum*
**Figure S2:** Distribution of expression levels based on Ct values from each reference gene. (A) GAPDH; (B) 18S; (C) ACTB; (D) HPRT1; (E) B2M. For statistical analyses, the one‐way ANOVA followed by the Tukey test was used to evaluate differences between the groups. Significant differences (*p* ≤ 0.05) are represented by *p < 0.05, **p < 0.01 and ***p < 0.001 (*n* = 6 per group).
**Figure S3:** Comparison of stability tests calculated by four computer programs between BALB/c and C57BL/6j. (A) GeNorm; (B) BestKeeper; (C) NormFinder; (D) RefFinder.
**Figure S4:** Dissociation curves (including melting temperatures) and 2% agarose gel electrophoresis of the amplification products of genes analyzed. 100 bp DNA Ladder was used; Blank represents no template control.
**Table S1:** Liver histopathological scoring system
**Table S2:** Information on the efficiency of the primers used in this work. Slope; E: Amplification efficiency in %; R2: Correlation efficiency and Melting temperature of each gene used.
**Table S3:** Percentage of larval recovery according to the administered dose. Values represent the percentage of larvae recovered relative to the total number of eggs inoculated in each experimental animal per group.

## Data Availability

The data that support the findings of this study are available from the corresponding author upon reasonable request.
