# Supplementary material for: Chronic Cholestatic Liver Disease Induced by Larval Ascariasis: Novel Insights Into Immune‐Mediated Pathogenesis and Hepatic Fibrosis in Mice
Source: FASEB J. 2026 May 17;40:e71912. doi: 10.1096/fj.202600771R (PMC13181307; doi:10.1096/fj.202600771R)
Supplement: Supplementary file 1 — Figure S1: Experimental design to evaluation of reference genes in the liver of BALB/c and C57BL/6j at the peak of infection of A. suum Figure S2: Distribution of expression levels based on Ct values from each reference gene. (A) GAPDH; (B) 18S; (C) ACTB; (D) HPRT1; (E) B2M. For statistical analyses, the one‐way ANOVA followed by the Tukey test was used to evaluate differences between the groups. Significant differences (p ≤ 0.05) are represented by *p < 0.05, **p < 0.01 and ***p < 0.001 (n = 6 per group). Figure S3: Comparison of stability tests calculated by four computer programs between BALB/c and C57BL/6j. (A) GeNorm; (B) BestKeeper; (C) NormFinder; (D) RefFinder. Figure S4: Dissociation curves (including melting temperatures) and 2% agarose gel electrophoresis of the amplification products of genes analyzed. 100 bp DNA Ladder was used; Blank represents no template control. Table S1: Liver histopathological scoring system Table S2: Information on the efficiency of the primers used in this work. Slope; E: Amplification efficiency in %; R2: Correlation efficiency and Melting temperature of each gene used. Table S3: Percentage of larval recovery according to the administered dose. Values represent the percentage of larvae recovered relative to the total number of eggs inoculated in each experimental animal per group. [file FSB2-40-e71912-s001.docx]

**SUPPLEMENTARY MATERIALS**

**Chronic Cholestatic Liver Disease Induced by Larval Ascariasis: Novel Insights into Immune-Mediated Pathogenesis and Hepatic Fibrosis in Mice**

Jorge Lucas Nascimento Souza^1,2^, Chiara Cássia Oliveira Amorim^1^, Ana Rafaela Antunes Porto^1^, Fernanda Rezende Souza^3^, Evelyn Ane Oliveira^3^, Izabela da Silva Oliveira^1^, Isabela de Brito Duval^1^, Andressa Mariana Saldanha-Elias^1^, Marcelo Eduardo Cardozo^1^, Ramayana Morais de Medeiros Brito^1^, Luisa Mourão Dias Magalhães^4^, Geovanni Dantas Cassali^3^, Neima Briggs^5^, Ricardo Toshio Fujiwara^1^, Remo Castro Russo^2^, Guilherme Grossi Lopes Cançado^6^, Lilian Lacerda Bueno^1*^

^1^Laboratory of Immunobiology and Control of Parasites, Department of Parasitology, Institute of Biological Sciences, Universidade Federal de Minas Gerais, Belo Horizonte, Brazil

^2^Laboratory of Pulmonary Immunology and Mechanics, Department of Physiology and Biophysics, Institute of Biological Sciences, Universidade Federal de Minas Gerais, Belo Horizonte, Brazil

^3^Laboratory of Comparative Pathology, Department of Pathology, Institute of Biological Sciences, Universidade Federal de Minas Gerais, Belo Horizonte, Brazil

^4^Laboratory of Immunology of Parasitic Interactions, Department of Parasitology, Institute of Biological Sciences, Universidade Federal de Minas Gerais, Belo Horizonte, Brazil

^5^Department of Internal Medicine (Infectious Diseases), Yale University School of Medicine, New Haven, Connecticut, United States

^6^Instituto Alfa de Gastroenterologia, Hospital das Clínicas, Universidade Federal de Minas Gerais, Brazil

***Author for correspondence:** Lilian Lacerda Bueno, Laboratory of Immunobiology and Control of Parasites, Department of Parasitology, Institute of Biological Sciences, Universidade Federal de Minas Gerais, Belo Horizonte, Brazil. Avenida Antônio Carlos 6627, Belo Horizonte, 31270-901, Minas Gerais, Brazil. E-mail: [lilacerdabueno@gmail.com](mailto:lilacerdabueno@gmail.com)

**Table of Contents**

| **Table. S1**………………….................................................................................................. **3**  **Table. S2**…………………...................................................................................................**4**  **Fig. S1**…………………….................................................................................................. **5**  **Fig. S2**…………………….................................................................................................. **5**  **Fig. S3**…………………….................................................................................................. **6**  **Fig. S4**…………………….................................................................................................. **7**  **Table. S3**………………….................................................................................................. **8**  **Table S1:** Liver histopathological scoring system     \| **Semiquantitative histopathological liver analysis** \| \| \| --- \| --- \| \| **Score** \| **Liver injury score** \| \| 0 \| Absence of inflammatory cells around hepatocytes, blood vessels, bile ducts and capillaries, absence of necrosis areas (absent) \| \| 1 \| A few regions of the liver parenchyma contain small inflammatory foci with reduced number of inflammatory cells, a few necrosis areas (mild) \| \| 2 \| Liver parenchyma contain inflammatory foci with moderate number of inflammatory cells, perivascular inflammatory infiltrate, as well as around the ducts, and small areas of necrosis scattered throughout the parenchyma (moderate) \| \| 3 \| Hepatic parenchyma often presents larger inflammatory foci, diffuse inflammatory infiltrate, abundant perivascular inflammation around the ducts, areas of necrosis scattered throughout the parenchyma (intense) \| |
| --- | --- | --- | --- | --- | --- | --- | --- | --- | --- | --- | --- | --- |

**Table S2. Information on the efficiency of the primers used in this work.** Slope; E: Amplification efficiency in %; R2: Correlation efficiency and Melting temperature of each gene used.

| **Gene** | **Slope** | **E(%)** | **R^2^** | **Melting temperature** |
| --- | --- | --- | --- | --- |
| **IL-12/p40** | -3,42 | 96,06 | 0,998 | 75,28 |
| **IFN-γ** | -3,49 | 93,43 | 0,993 | 72,32 |
| **IL-1-β** | -3,45 | 94,42 | 0,998 | 83,11 |
| **IL-6** | -3,45 | 94,92 | 0,994 | 79,89 |
| **IL-4** | -3,52 | 92,35 | 1,000 | 77,47 |
| **IL-5** | -3,16 | 107,23 | 0,989 | 74,56 |
| **IL-17** | -3,55 | 91,29 | 0,999 | 87,21 |
| **IL-10** | -3,43 | 95,68 | 0,988 | 82,22 |
| **TGF-β** | -3,22 | 104,44 | 1,000 | 84,23 |

**Fig. S1**


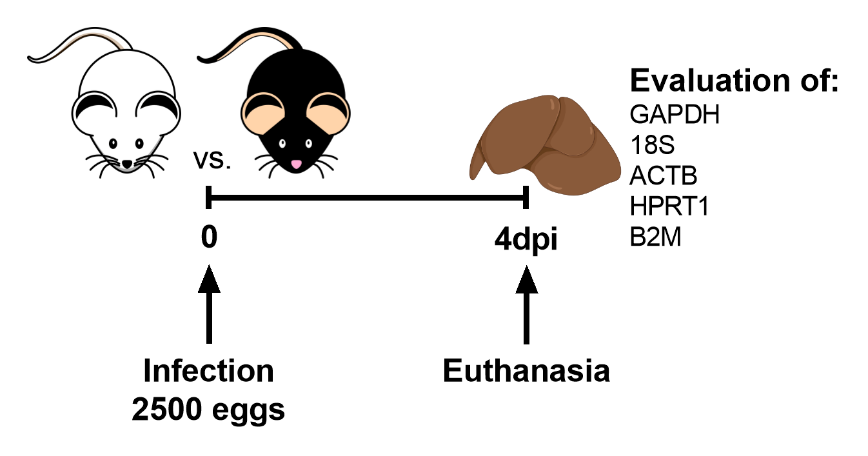


**Fig. S1. Experimental design to evaluation of reference genes in the liver of BALB/c and C57BL/6j at the peak of infection of *A. suum***

**Fig. S2.**


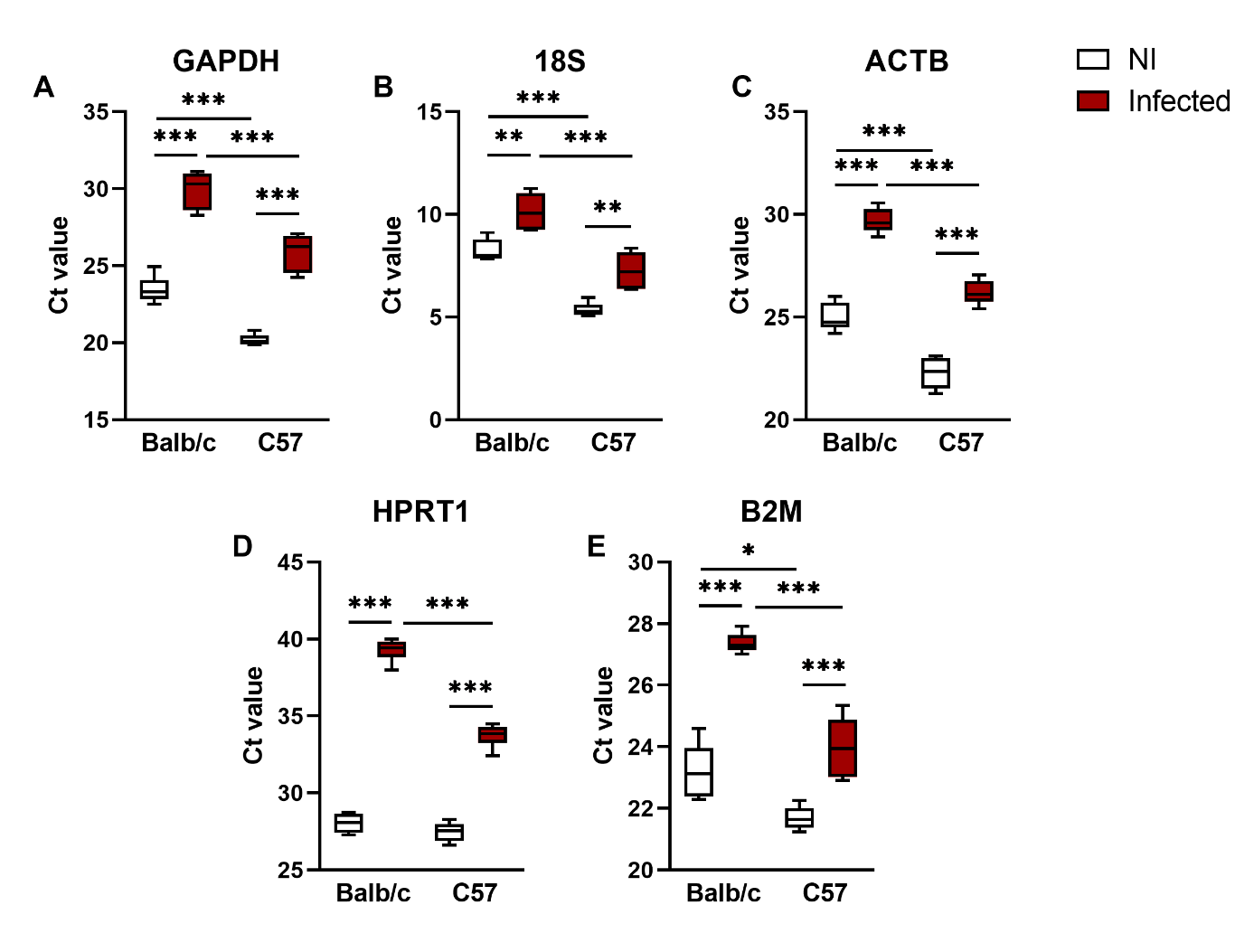


**Fig. S2. Distribution of expression levels based on Ct values from each reference gene.** (A) GAPDH; (B) 18S; (C) ACTB; (D) HPRT1; (E) B2M. For statistical analyses, the one-way ANOVA followed by the Tukey test was used to evaluate differences between the groups. Significant differences (p ≤ 0.05) are represented by *p<0.05, **p<0.01 and ***p<0.001 (n=6 per group).

**Fig. S3.**


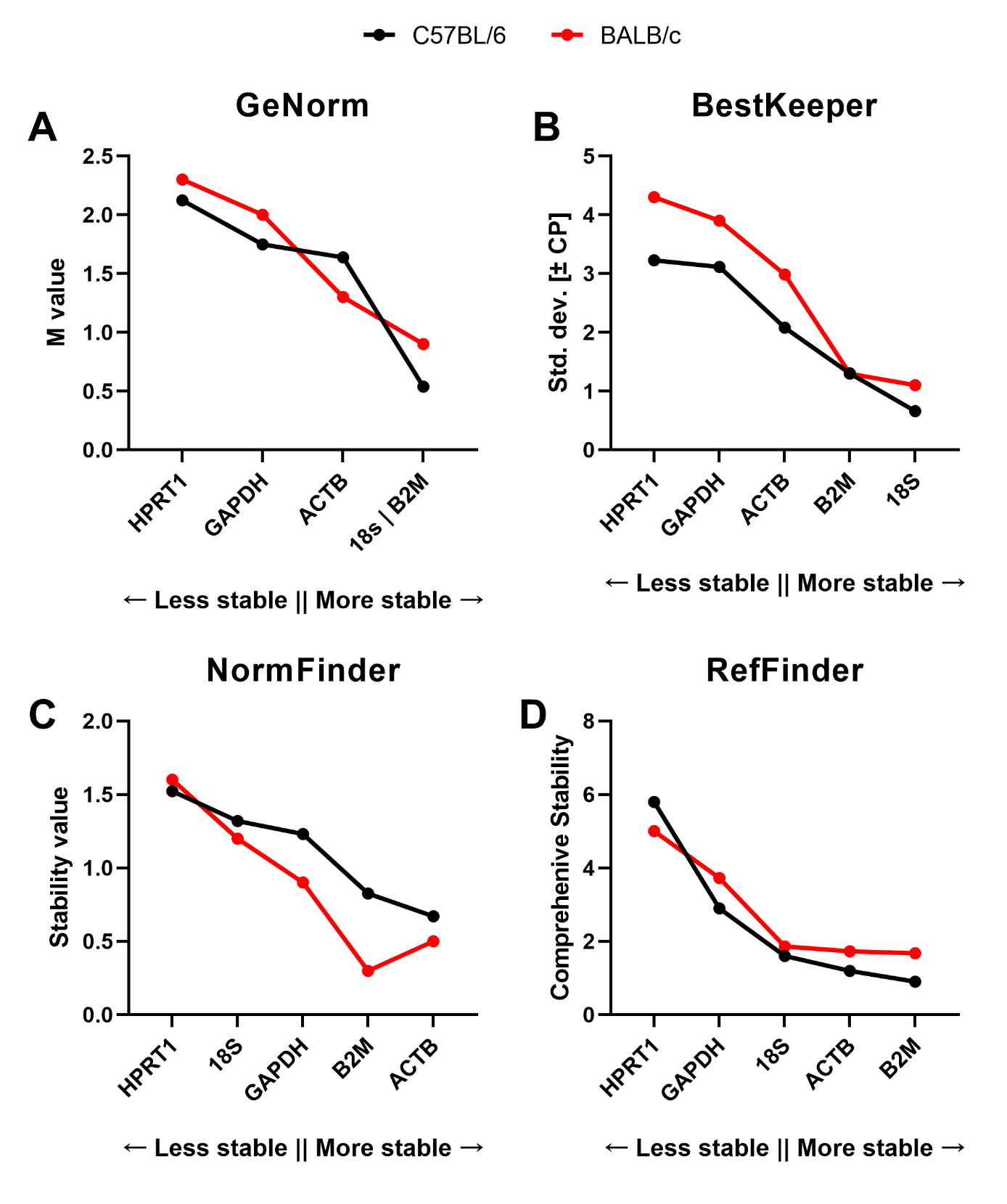


**Fig. S3. Comparison of stability tests calculated by four computer programs between BALB/c and C57BL/6j.** (A) GeNorm; (B) BestKeeper; (C) NormFinder; (D) RefFinder.

**Fig. S4**


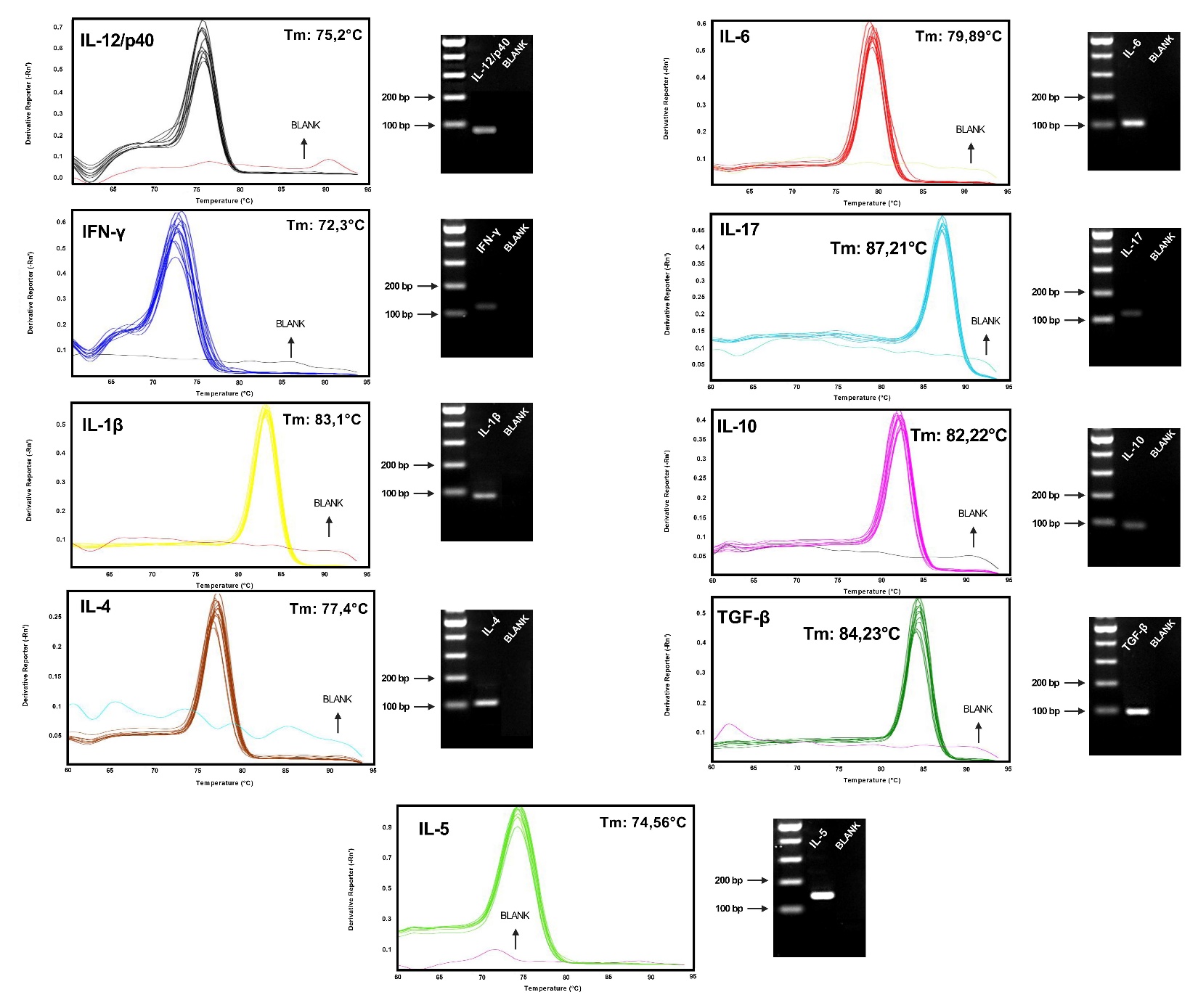


**Fig. S4. Dissociation curves (including melting temperatures) and 2 % agarose gel electrophoresis of the amplification products of genes analyzed.** 100 bp DNA Ladder was used; Blank represents no [template](https://www.sciencedirect.com/topics/biochemistry-genetics-and-molecular-biology/dna-template) control.

**Table S3. Percentage of larval recovery according to the administered dose.** Values represent the percentage of larvae recovered relative to the total number of eggs inoculated in each experimental animal per group.

| **Animal** | **SI 250** | **RE250** | **SI2500** | **RE2500** |
| --- | --- | --- | --- | --- |
| **A1** | 9,2% | 12,4% | 6,12% | 6,08% |
| **A2** | 11,2% | 13,6% | 6,44% | 6,28% |
| **A3** | 12% | 13,2% | 6,64% | 6,4% |
| **A4** | 17,6% | 18,8% | 6,96% | 7,52% |
| **A5** | 20% | 19,6% | 7,08% | 7,92% |
| **A6** | 22% | 20% | 7,28% | 8,12% |
| **A7** | 26,8% | 20,8% | 7,2% | 8,32% |
| **A8** | 27,6% | 21,2% | 7,56% | 8,8% |
| **Mean** | 18,30% | 17,45% | 6,91% | 7,43% |
